# Genetic Interactions between the Members of the SMN-Gemins Complex in *Drosophila*

**DOI:** 10.1371/journal.pone.0130974

**Published:** 2015-06-22

**Authors:** Rebecca M. Borg, Rémy Bordonne, Neville Vassallo, Ruben J. Cauchi

**Affiliations:** 1 Department of Physiology and Biochemistry, Faculty of Medicine and Surgery, University of Malta, Msida, Malta GC; 2 Institut de Génétique Moléculaire de Montpellier, CNRS-UMR5535, Université Montpellier 1 and 2, Montpellier, France; CINVESTAV-IPN, MEXICO

## Abstract

The SMN-Gemins complex is composed of Gemins 2–8, Unrip and the survival motor neuron (SMN) protein. Limiting levels of SMN result in the neuromuscular disorder, spinal muscular atrophy (SMA), which is presently untreatable. The most-documented function of the SMN-Gemins complex concerns the assembly of spliceosomal small nuclear ribonucleoproteins (snRNPs). Despite multiple genetic studies, the Gemin proteins have not been identified as prominent modifiers of SMN-associated mutant phenotypes. In the present report, we make use of the *Drosophila* model organism to investigate whether viability and motor phenotypes associated with a hypomorphic Gemin3 mutant are enhanced by changes in the levels of SMN, Gemin2 and Gemin5 brought about by various genetic manipulations. We show a modifier effect by all three members of the minimalistic fly SMN-Gemins complex within the muscle compartment of the motor unit. Interestingly, muscle-specific overexpression of Gemin2 was by itself sufficient to depress normal motor function and its enhanced upregulation in all tissues leads to a decline in fly viability. The toxicity associated with increased Gemin2 levels is conserved in the yeast *S*. *pombe* in which we find that the cytoplasmic retention of Sm proteins, likely reflecting a block in the snRNP assembly pathway, is a contributing factor. We propose that a disruption in the normal stoichiometry of the SMN-Gemins complex depresses its function with consequences that are detrimental to the motor system.

## Introduction

Spinal muscular atrophy (SMA) is a primarily early-onset neuromuscular disorder with hallmark features that include loss of spinal motor neurons as well as atrophy of the proximal limb and intercostal muscles. This devastating condition remains one of the most frequently inherited causes of infant mortality since current therapeutic options are, at best, palliative. In the majority of cases, SMA is the result of insufficient levels of the ubiquitously-expressed survival motor neuron (SMN) protein [1, 2]. SMN associates with Gemins 2–8 and Unrip to form the large macromolecular SMN-Gemins complex. Whilst this elaborate nine-membered complex is typical in humans, the simplest version composed of only SMN (Yab8p) and Gemin2 (Yip1p) is found in the fission yeast *Schizosaccharomyces pombe* whereas the fruit fly *Drosophila melanogaster* possesses a minimalistic complex counting only SMN, Gemin2, Gemin3 and Gemin5 amongst its constituents (reviewed in [3]).

The SMN-Gemins complex is indispensible for chaperoning the assembly of small nuclear ribonucleoproteins (snRNPs), which are crucial for pre-mRNA splicing (reviewed in [4–6]). The intricacies of this cytoplasmic process are now less opaque for Sm-class snRNPs. In essence, it involves the coupling of a heptameric ring of Sm proteins with small nuclear RNAs (snRNAs) to compose the snRNP core structure. Gemin5 is thought to identify nuclear-exported snRNAs [7], which it binds to via the N-terminal WD-repeat domain [8]. Following capture, snRNA-charged Gemin5 is thought to dock into the SMN-Gemins complex, most probably proximate to Gemin2, to deliver its cargo for Sm core assembly [9]. On the other hand, the majority of Sm proteins are recognised by Gemin2, which wraps itself around a crescent-shaped Sm pentamer. Importantly, the N-terminal tail of Gemin2 reaches into the snRNA-binding pocket on the pentamer to block their inclination for promiscuous RNA binding, presumably until they bind to snRNAs, which are their bona fide RNA substrates [10, 11]. The chaperoning of RNA and, eventually, RNP molecules as well as ATP breakdown during the assembly reaction, are probably fulfilled by DEAD-box RNA helicase Gemin3 [12–14], although biochemical and structural studies in this regard are lacking.

Whether a disruption in snRNP biogenesis and the consequential splicing defects, give rise to SMA is still a contentious issue, and should this be the case, the reasons why the motor unit is particularly vulnerable remain to be determined. Interestingly, recent studies have challenged the classical view of SMA pathophysiology entailing that spinal cord α-motor neurons are the primary cells affected and that muscle atrophy is the result of motor neuron defects. In this regard, corroborating an early investigation in *Drosophila* [15], recent studies on SMA mouse models demonstrated that restoring SMN expression pan-neuronally has minimal beneficial effects [16] whereas an increase in SMN levels in all tissues minus the central nervous system was sufficient for phenotypic rescue [17]. These and other findings (reviewed in [18]) question whether SMA is a cell-autonomous disease of motor neurons.

Known and unknown molecular pathways that are relevant to SMA pathology can be uncovered in an unbiased fashion via genetic approaches. In this regard, studies in patients [19–21] and, particularly, genome-wide screens in *Drosophila* [22, 23] as well as *C*. *elegans* [24] yielded several modifier genes. Surprisingly, in such studies, members of the Gemin family of proteins were not identified as prominent modifiers of *Smn*-associated mutant phenotypes although co-immunoprecipitation and proteomic approaches in *Drosophila* confirmed the interaction between SMN and key Gemin members [23, 25–28]. These findings might indicate that the study-specific screenable phenotype was not influenced by genes associated with snRNP biogenesis, the function that is most clearly associated with SMN. Hence, whilst such screens might inform on non-canonical SMN activities, they are limited in disclosing novel participants in snRNP assembly, a goal that becomes more relevant following the recent renaissance of the link between this canonical function and SMA’s signature features [29–34].

We have previously shown that in *Drosophila*, Gemin2, Gemin3 and Gemin5 co-localise with SMN [35, 36] and their loss-of-function specifically in the motor unit results in motor phenotypes that are similar to those described earlier for SMN [25, 37]. Whilst such findings indicate that these key members of the SMN-Gemins complex operate in a common pathway and this is corroborated by biochemical studies [38, 39], they fall short of confirming an interaction *in vivo*. In the present study, we make use of a hypomorphic Gemin3 mutant to probe whether the associated viability and/or motor phenotypes are enhanced by changes in the levels of SMN, Gemin2 and Gemin5 brought about by various genetic manipulations. We perform our investigations in muscle considering previous studies by us and others showing that this tissue has a greater requirement for SMN and Gemins compared to neurons in *Drosophila* [22, 25, 37]. For the first time, we show a modifier effect by all three SMN-Gemins complex constituents. Interestingly, overexpression of Gemin2 in a pan-muscular pattern in wild-type flies is by itself sufficient to depress normal motor behaviour and its enhanced upregulation in all tissues reduces viability. The latter phenotype is conserved in the yeast *S*. *pombe* in which we find that the cytoplasmic retention of Sm proteins, likely reflecting a block in snRNP biogenesis, contributes to toxicity. Our results lead us to speculate that an alteration in its stoichiometry or an imbalance in the levels of its constituent members destabilises the SMN-Gemins complex with consequences that are detrimental to the motor system and a severity that is dependent on the number of components altered simultaneously.

## Materials and Methods

### Fly Stocks

Flies were cultured on standard molasses/maizemeal and agar medium in plastic vials at an incubation temperature of 25°C. Wild-type strains were *y w* or *Oregon R* except where indicated. The *Gemin5* transposon insertion lines, *Gem5* ^*M*^ (PBac[3HPy+]rig^C063^), *Gem5* ^*P*^ (P[lacW]rig^k07917^), and *Gem5* ^*W*^ (P[PZ]rig^05056^), and the *Gemin2* chromosomal deletion (Df(3L)ED4782) were obtained from the Bloomington *Drosophila* Stock Center (NIH P40OD018537) at Indiana University, USA. The Gemin5 chromosomal deletion (Df(2R)exu1) was obtained from the *Drosophila* Genetic Resource Centre at the Kyoto Institute of Technology, Kyoto, Japan. The *Smn* ^*X7*^, *UAS*.*GFP-Smn* ^*FL*^, *UAS*.*GFP-Smn* ^*∆6*^, *UAS*.*Flag-Smn* ^*FL*^, *UAS*.*Smn-IR* ^*FL26B*^, *UAS*.*Smn-IR* ^*N4*^, and *UAS*.*Smn-IR* ^*C24*^ were generous gifts from Spyros Artavanis-Tsakonas (Harvard Medical School, Boston, Massachusetts, USA). The *UAS-Gem5*.*GFP*, *UAS-Gem5* ^*∆N*^, *UAS*.*Gem5-IR* ^*nanni*^, *UAS*.*Gem5-IR* ^*sacher*^, *UAS*.*Gem2* ^*FL*^, *UAS*.*Gem2* ^*∆N*^, *UAS*.*Gem2* ^*∆C*^ and *UAS*.*Gem2-IR* ^*gau*^ strains, were generated and characterised previously [37]. In this study, GAL4 lines utilised include *Mef2*-GAL4, *how*-GAL4, *da*-GAL4 and *1032*-GAL4, whose providence and expression pattern was described previously [25, 37]. Identification of the *Gem3* ^*BART*^ hypomorph entailed performing a re-mobilisation screen of the *Gem3* ^*∆N*^ mutant described in an earlier study [37]. Combination of alleles and transgenes was carried out according to standard genetic crossing schemes.

### Yeast Strains

The *S*. *pombe* strain carrying the *tdSmn* allele has been characterised previously [34]. Cells were grown on YES or minimal EMM2 medium with adequate supplements. To control plasmid expression, cells were cultured on EMM2-Leu-Ura plates and Thiamine was used to switch expression either ‘on’ (absence) or ‘off’ (presence). Standard methods were used for both growth and genetic manipulations [40].

### Plasmid constructions

A fragment encoding the fission yeast *Gemin2* gene (*yip11*) was amplified from the pTN-RC5 cDNA library (a gift from T. Nakamura, YGRC, Osaka, Japan). PCR products were cut with *Bam*HI and *Xma*I and cloned into the *S*. *pombe* pREP3∆ vector, a derivative of pREP3 harbouring the thiamine-repressible *nmt1* promoter [41]. The plasmid subsequently generated was *pREP3∆-SpGem2*. The *Smn* gene was PCR amplified from *pREFP42-Smn* plasmid [42] and cut in the same way as above to generate the pREP3∆-SpSmn plasmid. To construct the *pREP42-GFP*.*SmB* plasmid, the *S*. *pombe SmB* gene was amplified from the *pTN-RC5* cDNA library and subsequently cloned into the *Sal*I and *Bgl*II sites of the *pREP42GFP*.*N* vector. Plasmids were purified and confirmed by sequencing. Transformations were carried out according to standard techniques [40].

### Viability, Survival and Growth Assessment

In case of *S*. *pombe*, the drop test was utilised to compare viability and cell growth rate of different strains. Basically, cultures of comparable density were serially diluted, spotted on plates and incubated at 25°C for 5 days. For *Drosophila*, adult viability was calculated as the percentage of the number of adult flies with the appropriate genotype divided by the expected number for the cross. When indicated, a temperature of 29°C was utilised to amplify GAL4 activity. For survival analysis, adult flies were maintained in vials at a density of 15 to 20 flies per vial. The percentage number of flies alive at each time point measured was determined by dividing the number of flies still alive by the initial number of flies in the vial and multiplying the value by 100. During their adult lifespan, flies were transferred to new vials routinely.

### Puparial Axial Ratios

Puparial axial ratios were calculated by dividing the length by the width of the puparia, both of which were measured from still images.

### Flight Assay

In preparation for flight quantification, flies were first subjected to a ‘warm-up’ by inducing negative geotaxis in a new empty vial for 6 times. As detailed previously [37], the organisms were then introduced into the top of the Droso-Drome, which consisted of a 1L glass bottle coated with an alcohol-based sticky fluid, and divided into 4 sectors, of 5cm each, spanning a total height of 20cm. The number of flies in each sector was determined, divided by the total number of flies assessed and multiplied by 100 to generate the percentage number of flies per sector. The height or sector in which flies are distributed determines their flight ability. Flight assays were performed by the same experimentalist to minimise variability and allow comparability.

### Immunohistochemistry

Larval muscles and the male reproductive apparatus were dissected in 1x PBS, fixed in 4% paraformaldehyde in PBS and then washed in 1x PBS + 0.1% Triton X-100 (PBT). The tissues were then stained overnight at room temperature by mouse anti-GFP (1:1000; Roche Diagnostics Ltd.) antibodies. The next day, tissues were washed in PBT and stained overnight at room temperature with anti-mouse Alexa Fluor 488 or 546 secondary goat antibodies (1:50) and nuclear-staining Hoechst 33342 (1:500). Following a final wash in PBT, the samples were mounted in 90% glycerol with anti-fade. Epifluorescent pictures were acquired with an Optika B-600TiFL microscope (40x objective).

### Statistical methods

Significance was tested by the unpaired t-test or two-way ANOVA.

## Results

### Rescue analyses of insertion mutants attests to the functionality of a *Gemin5* transgene

**Fig 1:**
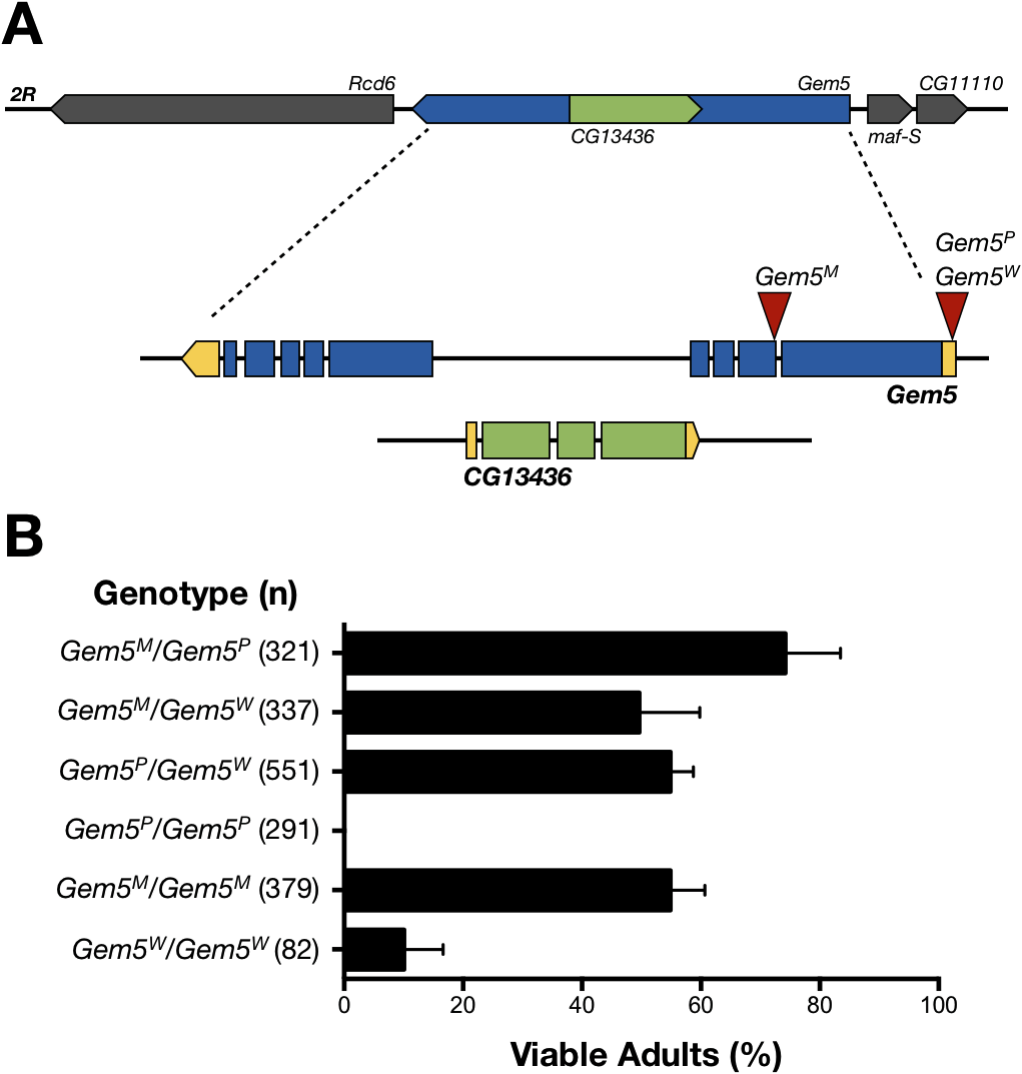
Rescue of lethality associated with homozygous and transheterozygous Gemin5 allelic combinations. (**A**) Genomic and genetic contexts of the *Gemin5* gene locus. The *CG13436* gene is nested within intron 4 of the *Gemin5* gene. Locations of two *P*-element insertions in the 5’ UTR, *Gem5* ^*P*^ and *Gem5* ^*W*^, as well as, *Gem5* ^*M*^, a PiggyBac insertion in exon 2 are indicated. (**B**) Analysis of the percentage number (mean ± S.E.M. of at least 4 independent experiments) of viable adults with ubiquitous transgenic expression of Gemin5 and the respective allelic combinations. Numbers of flies analysed are indicated in parenthesis. *Gem5* ^*M*^ homozygotes and transheterozygous mutant combinations all show adequate level of rescue. Rescue is absent and sub-optimal for *Gem5* ^*P*^ and *Gem5* ^*W*^ homozygotes, respectively.

The *Drosophila Gemin5* gene consists of 9 exons that encode for an approximately 138 kDa protein with a high conservation to its human counterpart especially within the WD repeat domain-rich N-terminus [37]. Gates et al. [43] reported that *Gemin5* transposon insertion mutants died in their majority as third instar larvae prior to developing moulting defects. However, the presence of a nested gene (*CG13436*) within intron 4 (**Fig 1A**), which might also be disrupted, raises questions about the specificity of the *Gemin5* mutants. Attempting at clarifying this issue as well as confirming the functionality of a *Gemin5* transgene, we performed rescue analysis on homozygous and transheterozygous allelic combinations. Via complementation crosses we first confirmed that the two mutants described by Gates et al. [43], including *Gem5* ^*P*^ and *Gem5* ^*W*^ each having a *P*-element insert in the 5’ UTR, retain their recessive lethality in trans to each other (*Gem5* ^*W*^ */Gem5* ^*P*^) and to chromosomal deficiencies that completely abolish the *Gemin5* gene amongst others (*Gem5* ^*W*^ */Df(2R)exu1*, *Gem5* ^*W*^ */Df(2R)exu2*, *Gem5* ^*P*^ */Df(2R)exu1*, and *Gem5* ^*W*^ */Df(2R)exu2*). In addition, *Gem5* ^*M*^, a new mutant with a PiggyBac insertion in the 2^nd^ exon was also lethal in the homozygous state (*Gem5* ^*M*^ */Gem5* ^*M*^) or when combined with *Gem5* ^*P*^ (*Gem5* ^*M*^ */Gem5* ^*P*^), *Gem5* ^*W*^ (*Gem5* ^*M*^ */Gem5* ^*W*^) or chromosomal deficiencies (*Gem5* ^*M*^ */Df(2R)exu1*, and *Gem5* ^*M*^ */Df(2R)exu2*) in a transheterozygous state. Ubiquitous expression of a *Gemin5* transgene driven by *da*-GAL4 rescued the lethality of both *Gem5* ^*M*^ and *Gem5* ^*W*^ homozygotes, although the degree of rescue was higher in the former compared to the latter (**Fig 1B**). No rescue was obtained for *Gem5* ^*P*^ homozygotes (*Gem5* ^*P*^ */Gem5* ^*P*^) exposing the influence of a non-specific mutation on its lethal phenotype. Transheterozygous combinations of all three alleles, including *Gem5* ^*M*^ */Gem5* ^*P*^, *Gem5* ^*M*^ */Gem5* ^*W*^, and *Gem5* ^*P*^ */Gem5* ^*W*^, were rescued to a similar degree, with the level of rescue being similar or higher to that of *Gem5* ^*M*^ homozygotes. These findings attest to the functionality of the Gemin5 transgene utilised, hence, allowing its use in downstream experiments.

### Sub-cellular Gemin5 expression pattern subsides on gene add-back in mutants

**Fig 2:**
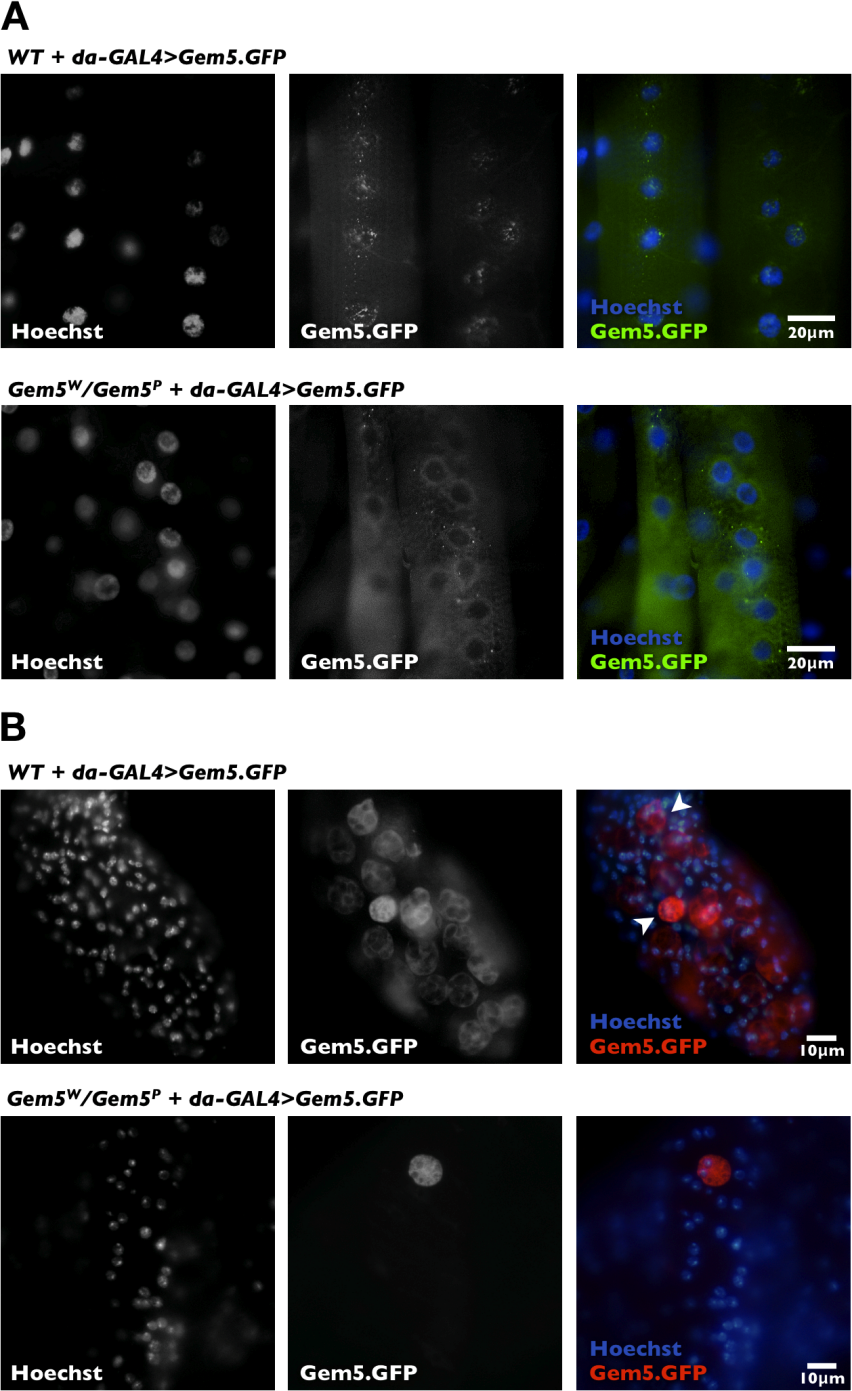
Sub-cellular Gemin5 expression pattern in rescued mutant flies. (**A**) Larval muscles of wild-type or mutant flies ubiquitously expressing a GFP-tagged Gemin5 fusion protein. Whereas Gemin5 overexpression induces supernumerary bodies that are largely nuclear, its expression in a mutant background reduces the number of foci, which are mostly restricted to the cytoplasm. (**B**) Tip of male accessary gland showing GFP expression in secondary cells is higher in flies overexpressing Gemin5 compared to rescued mutants. Secondary cells have a spherical appearance and multiple, large vacuoles (arrowhead).

In *Drosophila* ovaries, Gemin5 is enriched with other SMN-Gemins complex members and snRNPs in discrete cytoplasmic structures known as U bodies [35, 44]. SMN-Gemins complexes also tend to congregate in conspicuous nuclear bodies, known as gems [36, 45]. Consequently, we have recently reported that overexpression of a fluorescent reporter-tagged gene in a wild-type background results in the localisation of Gemin5 to supernumerary foci of variable size that are predominantly nuclear within the muscular tissue [37]. In this regard, we asked whether this expression pattern is maintained in rescued *Gemin5* mutants. We find that this is not the case; hence, we observe a decrease in the number of bodies that remain predominantly confined within the cytoplasm (**Fig 2A**). It is noteworthy that this expression pattern probably reflects that of the endogenous protein.

Human proteome studies have reported that Gemin5 expression is the highest in the gonads [46, 47]. Interestingly, we noticed that ubiquitous overexpression of GFP-tagged Gemin5 in wild-type flies results in the fusion protein being prominently enriched within the male reproductive apparatus, specifically the secondary cells of the accessory gland (**Fig 2B**). The key reproductive function of the accessory gland is the synthesis of seminal proteins that induce post-mating changes in females, including changes in egg laying, receptivity to courting males and sperm storage. Males have two accessory glands that are composed of a monolayer of secretory cells that can be divided into two morphologically- and biochemically-distinct cell types: flat, polygonally-shaped ‘main cells’ and large, spherical, vacuole filled ‘secondary cells’. Whilst main cells constitute the primary cell type of the accessary gland, secondary cells are only found at the distal tip of the gland where they are found dispersed among the main cells [48]. We note that similar to what we observed in muscle, in rescued Gemin5 mutants, expression levels diminished, hence, a lesser number of secondary cells were enriched with GFP-tagged Gemin5 fusion protein (**Fig 2B**).

### Identification and characterisation of a Gemin3 hypomorphic mutant

**Fig 3:**
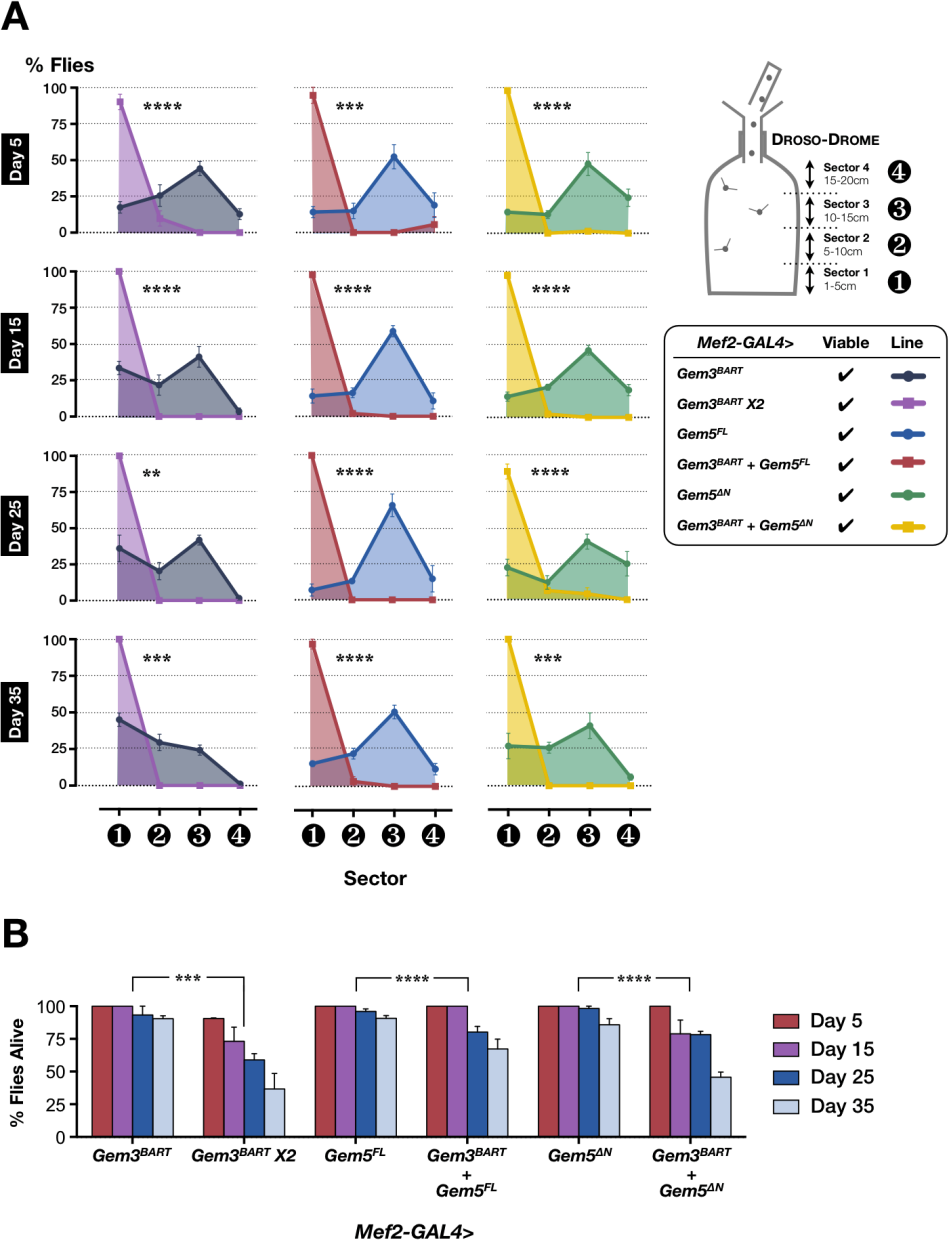
Overexpression of full-length or N-terminal truncated Gemin5 escalate flight and viability defects of a Gemin3 hypomorphic mutant. (**A**) A double but not a single dose of Gemin3 hypomorph, *Gem3* ^*BART*^, driven via the pan-muscular driver *Mef2*-GAL4 driver results in a high percentage of adult flies that are flight-impaired at all the time points measured (left panel). Overexpression of either full-length (middle panel) or N-terminally truncated Gemin5 (right panel) enhance the flight defects associated with the ectopic expression of *Gem3* ^*BART*^ in muscle tissues. Flight performance was determined via Droso-Drome runs, in which the height a fly falls indicates its flight capability. Fliers concentrate in the upper sectors whereas non-fliers drop to sector 1, the lowest sector. (**B**) Percentage number of flies alive assessed at different time points during adulthood. Muscle-restricted ectopic expression of two *Gem3* ^*BART*^ transgenes results in a statistically significant drop in adult fly survival compared to the expression of a single transgene. A similar decline in survival is obtained on overexpression of either full-length or N-terminally truncated Gemin5 in a *Gem3* ^*BART*^ background. In both (A) and (B) data presented are the mean ± S.E.M. of at least 4 independent experiments, and n ≥ 60 per genotype for each time point measured. Significance was tested by the unpaired t-test and two-way ANOVA in (A) and (B), respectively, and for all data, **p<0.01, ***p<0.001, and ****p<0.0001.

Pan-muscular overexpression of Gem3^∆N^, a truncated Gemin3 mutant lacking the helicase core, results in reduced viability, motor defects and flight muscle atrophy [25]. Recently, based on genetic evidence, we demonstrated that the Gem3^∆N^ mutant mimics a loss-of-function by presumably interfering at some level with the activity of the endogenous Gemin3 protein or its associated complex [37]. In this study, we screened a collection of randomly-inserted *Gem3* ^*∆N*^ lines and identified strains in which activation of *Gem3* ^*∆N*^ gene via the *UAS*/GAL4 system results in the induction of only low levels of the mutant. One particular transgene, *Gem3* ^*BART*^, was selected for subsequent experiments. Expression of this hypomorph in muscular tissue starting early during development (*Mef2-GAL4>Gem3* ^*BART*^) has no effect on both motor function and survival throughout adulthood (**Fig 3**). Motor function was measured via a flight assay in which the height a fly falls in a cylinder determines its flight performance. Hence, fliers are capable of holding onto the walls of upper sectors whereas flight-defective organisms drop to lower sectors. It is noteworthy that increasing the dose of Gem3^BART^ has drastic consequences. Indeed, flies with two copies of the mutant (*Mef2-GAL4>Gem3* ^*BART*^ *X2*) are flightless in their majority on day 5 post-eclosion, the earliest time point measured (**Fig 3A**). In this regard, 90% of the flies fall straight to the lowest sector. Flight ability declines rapidly so that on the remaining time points starting from day 15, all flies assayed were flight defective. Compared with flies having only one copy of *Gem3* ^*BART*^, those with a double dose were found to have an age-dependent progressive decline in adult survival (**Fig 3B**).

### Gemin5 upregulation or downregulation precipitate motor and viability defects associated with the Gem3^BART^ hypomorph

We wished to explore whether changes in the levels of SMN-Gemins complex members accelerate the phenotypic spectrum of the *Gem3* ^*BART*^ hypomorph. To this aim, we overexpressed Gemin5 with Gem3^BART^ in muscle tissues. We note that although full-length Gemin5 overexpression alone (*Mef2-GAL4>Gem5* ^*FL*^) has no negative influence, when in combination with *Gem3* ^*BART*^ (*Mef2-GAL4>Gemin3* ^*BART*^ *+ Gem5* ^*FL*^), flies exhibit both flight and viability defects (**Fig 3A**). Interestingly, similar defects can be induced if a Gemin5 transgene lacking its WD domain-rich N-terminus (*Gem5* ^*∆N*^) is used instead of a full-length Gemin5 transgene. This indicates that the C-terminus of Gemin5 is sufficient to destabilise the SMN-Gemins complex in the presence of Gem3^BART^.

**Fig 4:**
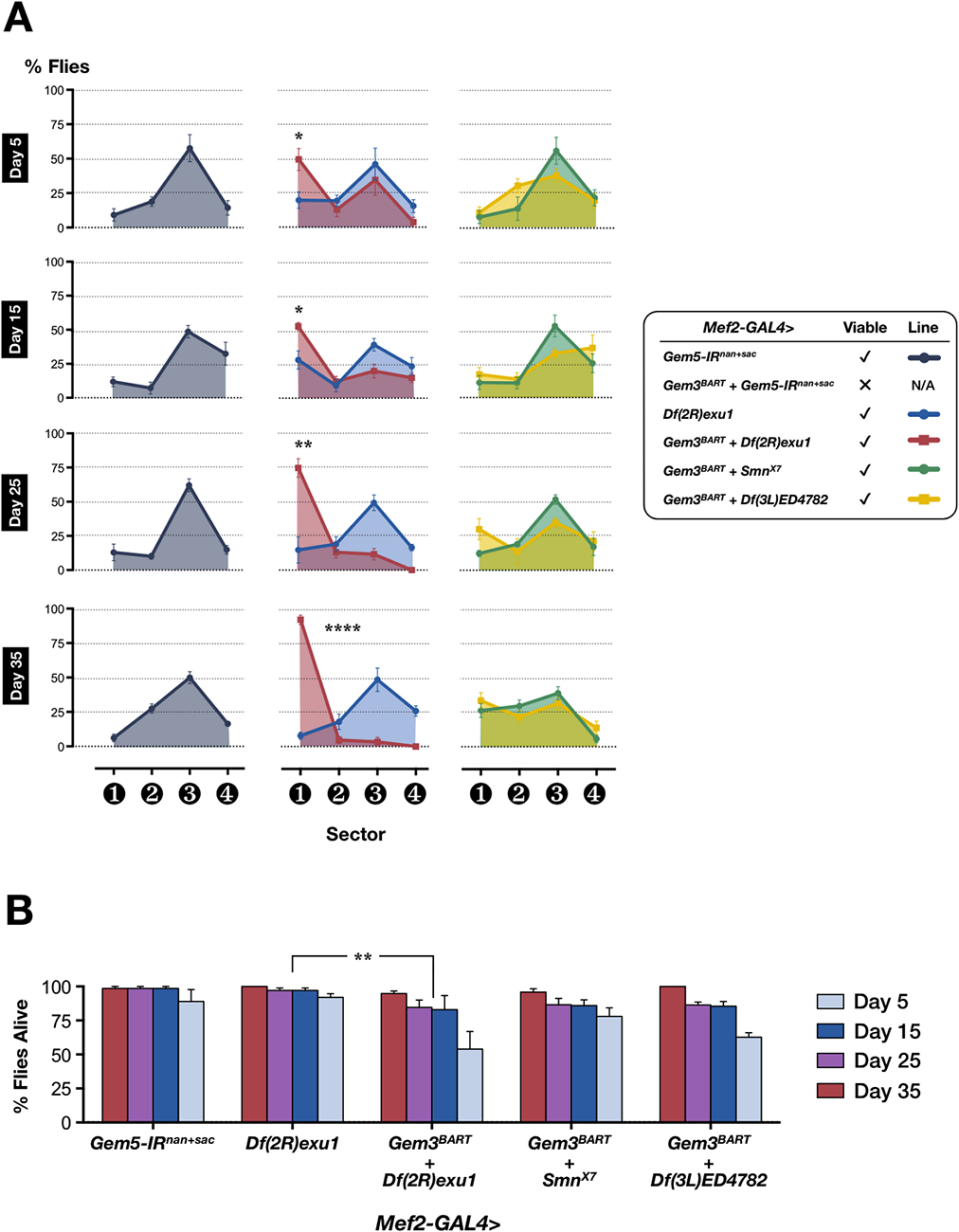
In combination with *Gem3* ^*BART*^, Gemin5 knockdown triggers lethality whereas a reduction in the gene copy number of Gemin5 provokes impaired flight. (**A**) Pan-muscular Gemin5 knockdown alone has no effect on flight performance (left panel). In a heterozygous Gemin5 deficient background brought about by a chromosomal deletion (*Df(2R)exu1* **)**, the hypomorphic *Gem3* ^*BART*^ motor phenotype becomes apparent on the day 5 time point and intensifies with age (middle panel). In this respect, the percentage of non-fliers in sector 1 increases significantly with age. In the heterozygous state, *Smn* (*Smn* ^*X7*^) or *Gemin2* deletion (*Df(3L)ED4782*) has no negative influence on *Gem3* ^*BART*^ (right panel). (**B**) Adult viability is significantly negatively impacted when a *Gemin5* chromosomal deletion is coupled with *Gem3* ^*BART*^. Gemin5 knockdown alone or *Gem3* ^*BART*^ in combination with *Smn* ^*X7*^ induce only a mild decrease in viability throughout adulthood. Compared to these genotypes, a moderate (but not severe) decrease in adult viability is seen in flies with both *Gemin3* ^*BART*^ and a chromosomal deletion that eliminates *Gemin2* (*Df(3L)ED4782*). In both (A) and (B) data presented are the mean ± S.E.M. of at least 4 independent experiments, and n ≥ 60 per genotype for each time point measured. Significance was tested by the unpaired t-test and two-way ANOVA in (A) and (B), respectively, and for all data, *p<0.05, **p<0.01, and ****p<0.0001.

We next questioned whether the *Gem3* ^*BART*^ phenotype would also be enhanced if Gemin5 levels were reduced. Augmented Dicer-2 levels were reported to enhance Gemin5 knockdown leading to phenotypic consequences [37]. However, in the absence of elevated Dicer-2 levels, Gemin5 knockdown in muscles (Mef2-GAL4>*Gem5-IR* ^*nan+sac*^) is uneventful (**Fig 4**). Importantly, flies with a pan-muscular Gemin5 knockdown coupled with the ectopic expression of *Gem3* ^*BART*^ (*Mef2-GAL4>Gem3* ^*BART*^ *+ Gem5-IR* ^*nan+sac*^) were not adult viable. A subtler reduction in Gemin5 levels through a reduction in gene copy number via a chromosomal deletion (*Df(2R)exu1*) was sufficient to expose the motor and viability defects intrinsic to the *Gem3* ^*BART*^ hypomorph. In this respect, muscle-specific *Gem3* ^*BART*^ expression in a heterozygous Gemin5 deficient background (*Mef2-GAL4>Gem3* ^*BART*^ *+ Df(2R)exu1*) gave rise to an age-progressive decline in flight ability starting from day 5 post-eclosion (**Fig 4A**). Viability was also significantly affected (**Fig 4B**). In contrast to what we observed for Gemin5, a background with a heterozygous deficiency of either *Smn* (*Mef2-GAL4>Gem3* ^*BART*^ *+ Smn* ^*X7*^) or *Gemin2* (*Mef2-GAL4>Gem3* ^*BART*^ *+ Df(3L)ED4782*) does not enhance the *Gem3* ^*BART*^ hypomorphic phenotype. Overall, these findings are suggestive of a genetic interaction between *Gemin3* and *Gemin5*.

### The Gem3^BART^ ectopic expression phenotype is enhanced by changes in SMN levels

**Fig 5:**
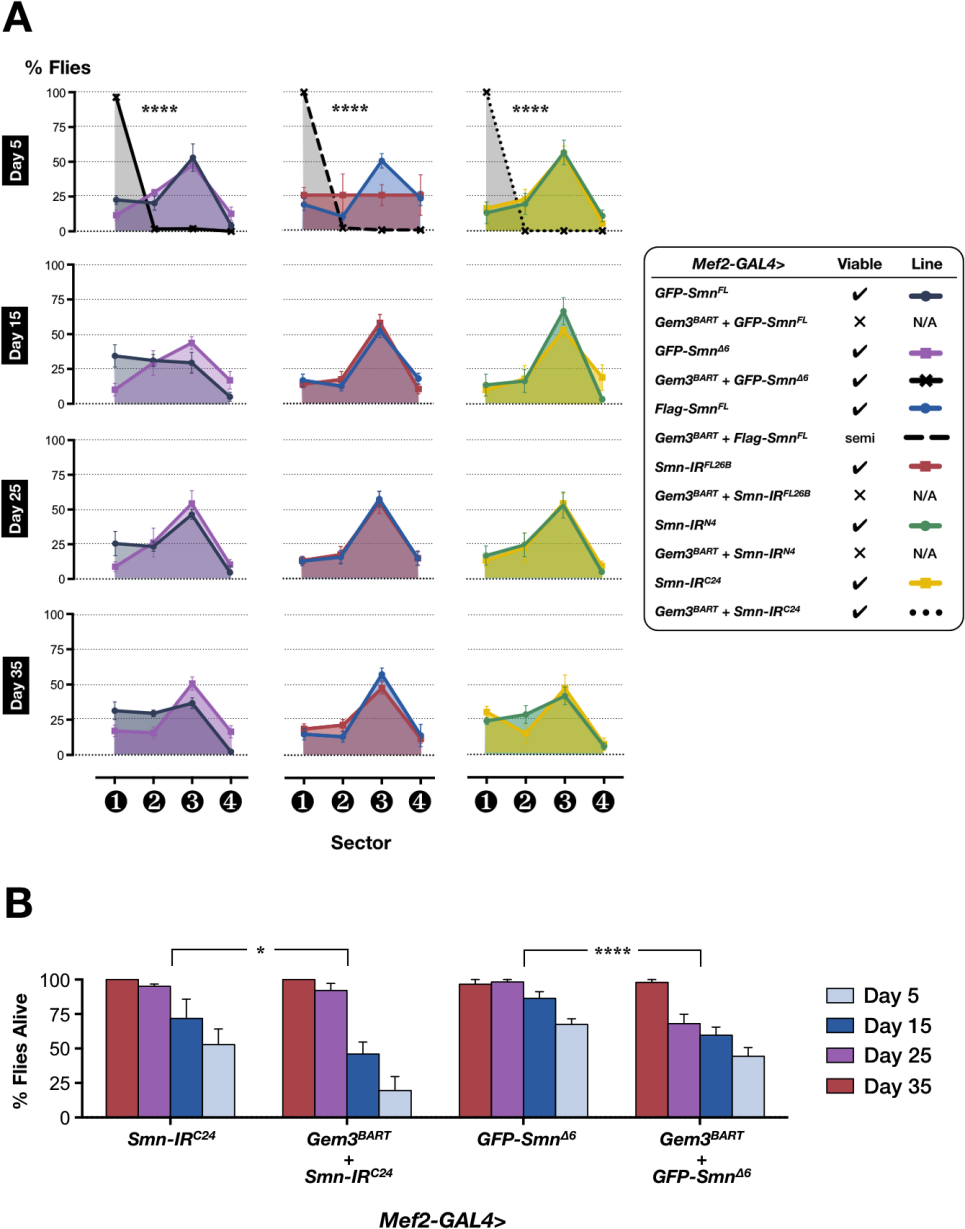
Augmentation or attenuation of SMN levels expose the motor and viability defects associated with the *Gem3* ^*BART*^ hypomorph. (**A**) Left panel: Overexpression of full-length SMN (*GFP-Smn* ^*FL*^) or a version lacking the region hosting the YG box (*GFP-Smn* ^*∆6*^) in muscle tissue is by itself inconsequential. However, in combination with *Gem3* ^*BART*^, flies exhibit either lethality (*Mef2-GAL4>Gem3* ^*BART*^ *+ GFP-Smn* ^*FL*^) or flightlessness (*Mef2-GAL4>Gem3* ^*BART*^ *+ GFP-Smn* ^*∆6*^). Statistical significance was determined for differences between the *Mef2-GAL4>Gem3* ^*BART*^ *+ GFP-Smn* ^*∆6*^ genotype, and the control *Mef2-GAL4>GFP-Smn* ^*∆6*^ genotype. Middle panel: Transgenic increase (*Mef2-GAL4>Flag-Smn* ^*FL*^) or decrease (*Mef2-GAL4>Smn-IR* ^*FL26B*^) in SMN levels, alone, has no impact on motor behaviour whereas in combination with *Gem3* ^*BART*^, the end-result is flight impairment (*Mef2-GAL4>Gem3* ^*BART*^ *+ Flag-Smn* ^*FL*^) and lethality (*Mef2-GAL4>Gem3* ^*BART*^ *+ Smn-IR* ^*FL26B*^), respectively. Statistical significance was determined for differences between the *Mef2-GAL4>Gem3* ^*BART*^ *+ Flag-Smn* ^*FL*^ genotype, and the control *Mef2-GAL4>Flag-Smn* ^*FL*^ genotype. Right panel: *Smn* knockdown, alone, through either targeting the N-terminus (*Mef2-GAL4>Smn-IR* ^*N4*^) or the C-terminus (*Mef2-GAL4>Smn-IR* ^*C24*^) has no obvious effect on flight behaviour, but it enhances the *Gem3* ^*BART*^ phenotype leading to lethality in *Mef2-GAL4>Gem3* ^*BART*^ *+ Smn-IR* ^*N4*^ flies or flight defects in *Mef2-GAL4>Gem3* ^*BART*^ *+ Smn-IR* ^*C24*^ flies. Statistical significance was determined for differences between the *Mef2-GAL4>Gem3* ^*BART*^ *+ Smn-IR* ^*C24*^ genotype, and the control *Mef2-GAL4>Smn-IR* ^*C24*^ genotype. (**B**) Compared to controls, a statistically significant drop in adult viability throughout adulthood is observed in flies with pan-muscular ectopic expression of Gem3^*BART*^ and either C-terminal targeted SMN knockdown (*Mef2-GAL4>Gem3* ^*BART*^ *+ Smn-IR* ^*C24*^) or overexpression of the SMN^*∆6*^ variant (*Mef2-GAL4>Gem3* ^*BART*^ *+ GFP-Smn* ^*∆6*^). In both (A) and (B) data presented are the mean ± S.E.M. of at least 4 independent experiments, and n ≥ 100 per genotype for each time point measured. Significance was tested by the unpaired t-test and two-way ANOVA in (A) and (B), respectively, and for all data, *p<0.05, and ****p<0.0001.

In *Drosophila*, Grice and Liu [49] reported that although SMN overexpression does not influence viability, it affects development leading to an alteration in both brain growth and the timing of cell differentiation in the testis. We asked whether the upregulation of SMN in muscle tissue has an effect on motor behaviour and, to this end, we found none whatsoever using two commonly used full-length *Smn* transgenes (*GFP-Smn* ^*FL*^ and *Flag-Smn* ^*FL*^) [22, 23] or a version lacking the region hosting the YG box domain (*GFP-Smn* ^*∆6*^) [22], a highly-conserved domain required for SMN oligomerisation (reviewed in [1]) (**Fig 5A**). However, in combination with *Gem3* ^*BART*^, a surplus of SMN results in lethality with few escapers (*Mef-GAL4> Gem3* ^*BART*^ *+ Flag-Smn* ^*FL*^) exhibiting severe flight defects (**Fig 5A**). Interestingly, overexpression of SMN^∆6^ had a lesser impact, hence, flies expressing both SMN^∆6^ and Gem3^BART^ in muscle are developmentally viable but in their majority they are flightless. Observation of adult flies with this genotype (*Mef-GAL4>Gem3* ^*BART*^ *+ GFP-Smn* ^*∆6*^) shows that compared to controls they experience a significant decline in survival throughout adulthood (**Fig 5B**).

We wished to determine if reduced SMN levels beyond the elimination of one gene copy (above) also enhance the interference of Gem3^BART^. The Artavanis-Tsakonas laboratory has recently generated RNA interference (RNAi) transgenic constructs targeting the full-length (*Smn-IR* ^*FL26B*^), amino-terminal (*Smn-IR* ^*N4*^) or carboxyl-terminal (*Smn-IR* ^*C24*^) portion of the *Smn* transcript. The authors show that driven by a strong pan-muscular driver (*how*-GAL4), RNAi-dependent knockdown of SMN induces neuromuscular junction and viability defects with the *Smn-IR* ^*N4*^ transgene displaying the most severe phenotype followed by *Smn-IR* ^*C24*^ and *Smn-IR* ^*FL26B*^ in that order [22]. In the present study, we show that when driven by a milder pan-muscular driver (*Mef2*-GAL4), there is no obvious loss of flight performance (**Fig 5A**). However, all three RNAi transgenes were found to enhance *Gem3* ^*BART*^-induced disruption. In this respect, in combination with *Gem3* ^*BART*^, the *Smn-IR* ^*N4*^ or *Smn-IR* ^*FL26B*^ transgenes induce lethality whereas the *Smn-IR* ^*C24*^ transgene triggers both motor and adult viability defects with respect to the appropriate controls (**Fig 5**). Overall, these findings are a clear indication of an *in vivo* interaction between *Smn* and *Gemin3*.

### Gemin2 overexpression affects motor function and in combination with Gem3^BART^ leads to lethality

**Fig 6:**
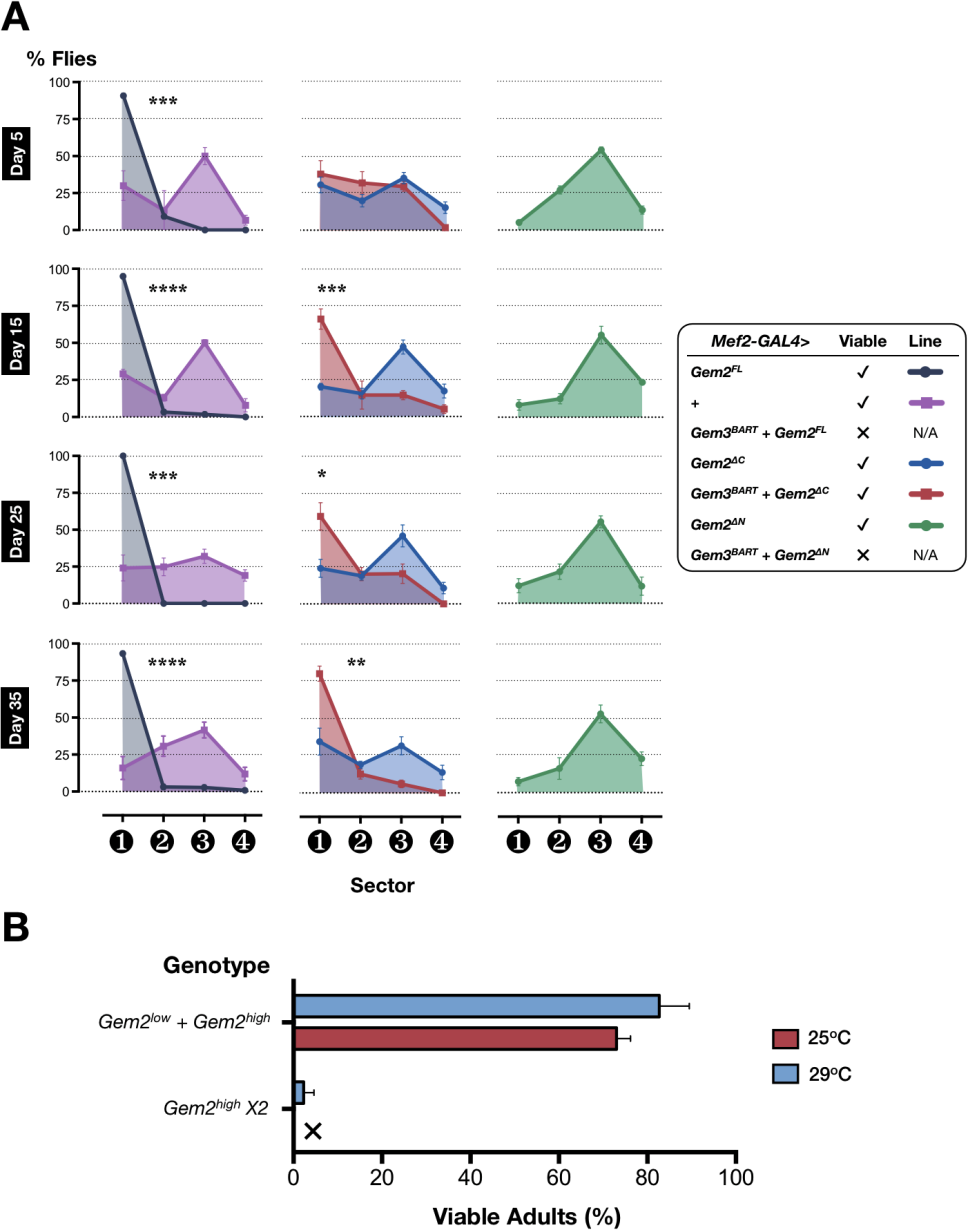
Gemin2 upregulation by itself is deleterious to viability as well as motor function and enhances the *Gem3* ^*BART*^ phenotype. (**A**) Left panel: Compared to the control genotype (*Mef2-GAL4/+*), pan-muscular overexpression of full-length Gemin2 (*Mef2-GAL4>Gem2* ^*FL*^) impairs flight early on during adulthood. In combination with *Gem3* ^*BART*^, Gemin2 overexpression (*Mef2-GAL4>Gem3* ^*BART*^ *+ Gem2* ^*FL*^) is lethal. Middle panel: Overexpression of the N-terminus of Gemin2 (*Mef2-GAL4>Gem2* ^*∆C*^) is uneventful with regards to motor function though when coupled with Gem3^*BART*^ (*Mef2-GAL4>Gem3* ^*BART*^ *+ Gem2* ^*∆C*^), it induces a progressive age-dependent decline in flight performance starting at day 15 post-eclosion. Right panel: Overexpression of the C-terminus of Gemin2 alone (*Mef-GAL4>Gem2* ^*∆N*^) has no effect on flight behaviour. In combination with *Gem3* ^*BART*^ (*Mef-GAL4>Gem3* ^*BART*^ *+ Gem2* ^*∆N*^), it triggers lethality. Data presented are the mean ± S.E.M. of at least 4 independent experiments, and n ≥ 60 per genotype for each time point measured. Significance was tested by the unpaired t-test, and for all data, *p<0.05, ***p<0.001, and ****p<0.0001. (**B**) Expression of a low expressing in combination with a high expressing full-length *Gemin2* transgene in all tissues via the *1032*-GAL4 driver has a marginal impact on adult viability. The expression of two high expressing Gemin2 transgenes results in a dramatic reduction in adult viability at a culture temperature of 25°C, and leads to lethality at culture temperatures associated with maximal GAL4 activity (29°C). Data presented are the mean ± S.E.M. of at least 4 independent experiments, and n ≥ 100 per genotype at either culture temperature.

**Fig 7:**
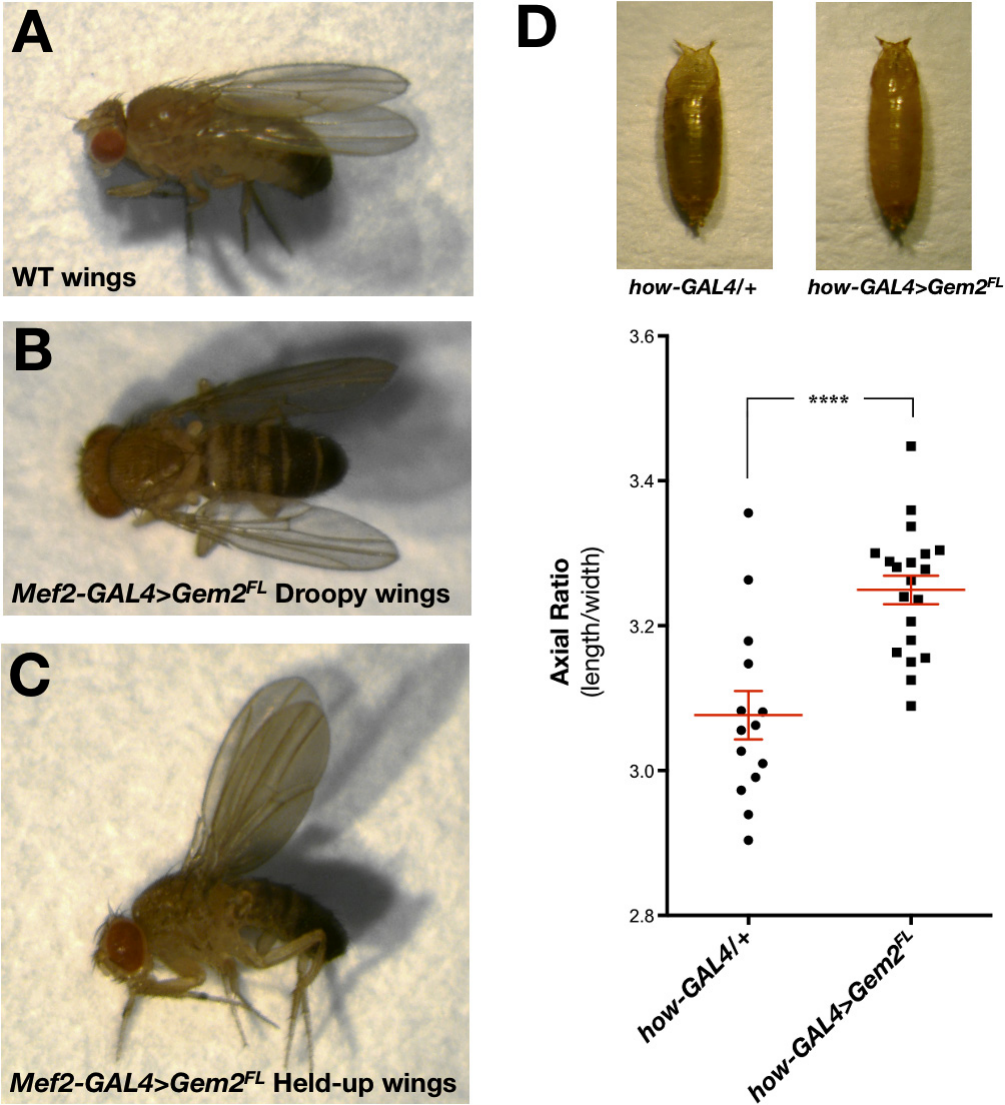
Gemin2 overexpressors display puparial and wing postural defects. Compared to wild-type flies, which have a dorsal wing posture (**A**), flies with a pan-muscular overexpression of full-length Gemin2 (*Mef2-GAL4>Gem2* ^*FL*^) present with either droopy (**B**) or held-up (**C**) wings. (**D**) *Top*, Puparia of flies with a strong mesodermal overexpression of Gemin2 (*how-GAL4>Gem2* ^*FL*^) and the GAL4 driver control (*how-GAL4/+*). *Bottom*, Chart showing that Gemin2 overexpressors (*how-GAL4>Gem2* ^*FL*^) have a significantly larger puparial axial ratio when compared to controls (*how-GAL4/+*). The mean is marked by a horizontal line running through the data points and error bars are ± S.E.M. (****p<0.0001; *how-GAL4/+*, n = 14; *how-GAL4>Gem2* ^*FL*^, n = 20).

In view of our findings on the genetic relationship between *Gemin3* and *Gemin5* as well as that between *Gemin3* and *Smn*, we next probed for an *in vivo* association between *Gemin3* and *Gemin2*, which is the only SMN-Gemins complex member with the most phylogenetically conserved sequence and domain structure [3]. Surprisingly, when overexpressed in muscle starting from early development (*Mef2-GAL4>Gem2* ^*FL*^), full-length Gemin2 is by itself detrimental, hence leading to motor defects early on during adulthood (**Fig 6A**). Furthermore, adult flies with this genetic manipulation exhibit different wing posture phenotypes, including droopy and held-up wings (**Fig 7A–7C**) when compared to controls in which wings typically run dorsal and parallel to the body. Survival of adult flies does not decline with age (data not shown). When Gemin2 expression is driven by a strong mesodermal driver (*how*-GAL4), flies failed to contract adequately during pupariation, consequently giving rise to significant differences in the puparial axial ratios when compared to the control genotype (**Fig 7D**), a phenotype we described previously following disruption of SMN or Gemin3 [25, 37].

Endeavouring to answer which Gemin2 domains are responsible for the negative effect on flight performance, we overexpressed N-terminal (*Mef2-GAL4>Gem2* ^*∆N*^) or C-terminal (*Mef2-GAL4>Gem2* ^*∆C*^) truncated versions of Gemin2 in muscle tissues. It is noteworthy that neither is consequential, and accordingly, flies with the respective genotype are good fliers at all measured time points during their adult life (**Fig 6A**). This finding suggests that both the N- and C-terminus of Gemin2 are required to induce flight defects. Subsequently, we queried what happens if full-length or truncated *Gemin2* is overexpressed together with *Gem3* ^*BART*^ in the same tissues. We observe that full-length Gemin2 or an N-terminal deletion (*Gem2* ^*∆N*^) induced lethality in *Gem3* ^*BART*^ flies. However, in combination with *Gem3* ^*BART*^, a *Gemin2* transgene lacking the C-terminus (*Gem2* ^*∆C*^) has lesser drastic consequences. Therefore, although *Mef-GAL4>Gem3* ^*BART*^ *+ Gem2* ^*∆C*^ flies were adult viable, they developed progressive age-dependent motor defects (**Fig 6A**). No effect on survival with age progression was observed (data not shown). Finally, we also demonstrate that when coupled with *Gem3* ^*BART*^, but not singularly, RNAi-induced reduction in Gemin2 levels has a negative influence on both motor function and adult viability with the severity of the latter phenotype depending on the level of Gemin2 knockdown (**S1 Fig**). On balance, these results constitute sufficient evidence of a genetic interaction between *Gemin2* and *Gemin3*.

### Toxicity of Gemin2 overexpression is conserved in *S*. *pombe*

We set out to further investigate the toxicity of Gemin2 overexpression considering that, to our knowledge, this phenomenon has not been previously reported. First, we determined the consequences of Gemin2 upregulation in all *Drosophila* tissues starting early during development. Minor effects on development were observed when a combination of two *Gemin2* transgenes, one that is low-expressing and another that is high-expressing, are driven by a mild ubiquitously-expressing GAL4 driver (**Fig 6B**). However, the use of two highly expressing *Gemin2* transgenes induces a major impact on adult viability. In this regard, only few escapers were counted when flies developed at a temperature of 25°C and none were observed when flies were cultured at a temperature of 29°C to allow for maximal GAL4 activity (**Fig 6B**). In combination with our previous report showing a similar deleterious effect on a global RNAi-induced knockdown [37], these findings indicate that ubiquitous Gemin2 protein levels influence adult viability.

**Fig 8:**
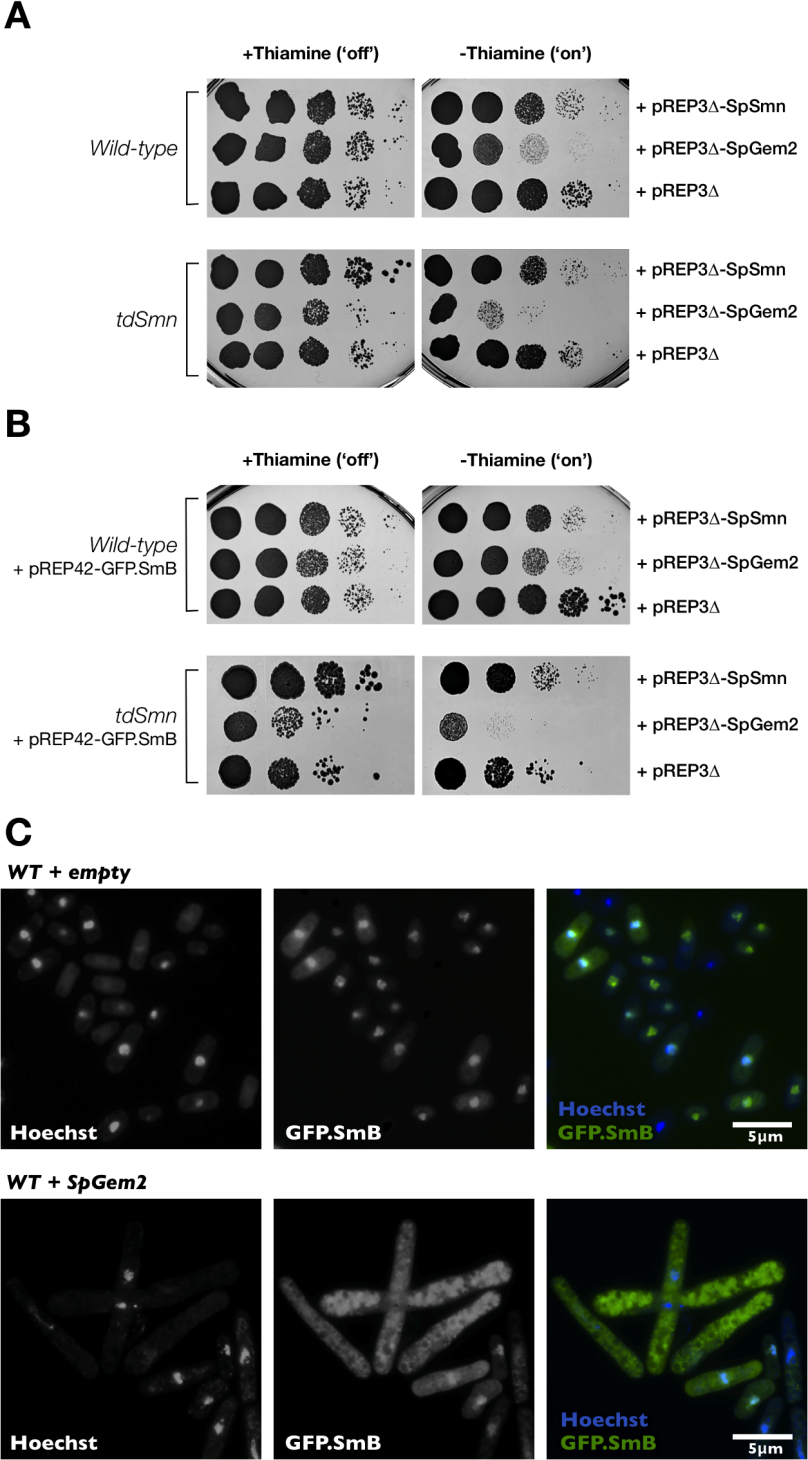
In *S*. *pombe*, Gemin2 overexpression affects cell viability through retention of Sm proteins in the cytoplasm. (**A**) Wild-type or *tdSmn* cells were transformed with a plasmid carrying the *S*. *pombe Smn* gene, a plasmid carrying the *S*. *pombe Gemin2* gene or with the empty pREP3∆ vector. Cultures of comparable density were then serially diluted, spotted on EMM2-Leu plates in the presence (expression is repressed) or absence (expression is induced) of Thiamine and incubated at 25°C for 5 days to test for their growth ability. In a wild-type or *tdSmn* background, growth defects are induced by upregulation of SpGem2 but not SpSMN. (**B**) Wild-type or *tdSmn* cells were transformed with a plasmid carrying GFP.SmB in combination with the plasmids indicated on the right. Cultures of comparable density were then serially diluted, spotted on EMM2-Leu-Ura- plates in the presence (expression is repressed) or absence (expression is induced) of Thiamine and incubated at 25°C for 5 days to examine their growth ability. The growth defect of either wild-type or *tdSMN* cells overexpressing Gemin2 is not complemented by an increase in the levels of SmB. *tdSMN* cells overexpressing both SmB and SpSMN, as expected, grew better than the control. However, the growth of wild-type cells with the same genetic modification was marginally inferior. (**C**) In GFP.SmB-expressing wild-type cells, SmB is predominantly localised to the nucleus. On upregulation of Gemin2, SmB accumulates in the cytoplasm and cells exhibit an elongated phenotype.

Subsequently, we asked whether Gemin2 overexpression is also detrimental in other model organisms, and to this end, we focused on *S*. *pombe*, which has been shown to be an excellent system to model human diseases [50]. In particular, we have previously demonstrated that cells carrying a temperature-degron *Smn* (*tdSmn*) allele mimic snRNP assembly and splicing defects observed in SMN deficient metazoan cells [34]. Wild-type and *tdSmn* cells were transformed with a plasmid carrying *SpGem2* (*yip11*) under the control of a very strong *nmt1* promoter. Cell cultures of comparable density were subjected to a drop test to investigate their ability to grow at 25°C for 5 days. In case of the *tdSmn* allele, at a temperature of 25°C, the function of SMN in snRNP assembly is already disrupted [34]. Compared to control (empty plasmid), *SpGem2* overexpressors displayed pronounced growth defects in either a wild-type or *tdSmn* background (**Fig 8A**). The same cannot be said for SMN. Thus, corroborating our results in *Drosophila* (above), overexpression of *SpSmn* had no negative influence on the growth rate of wild-type cells whereas, as expected, it improved growth when overexpressed in a *tdSmn* background.

### Mechanism of toxicity associated with Gemin2 overexpression involves cytoplasmic Sm protein retention

Finally, we were resolved to gain some hints on the mechanism through which abnormal concentrations of Gemin2 are toxic to cell viability. It can be hypothesised that overexpression of a member could disrupt a multiprotein complex into non-functional subassemblies. Alternatively, increased quantities of Gemin2 could compete for limiting amounts of Sm proteins, thereby reducing their presence in other molecules including snRNPs. In both hypotheses, it is envisaged that Sm proteins are retained in the cytoplasmic compartment because they are not coupled with their snRNA substrates, a requirement for their import into the nucleus where they function [51].

To investigate the location of Sm proteins in Gemin2 overexpressors, we double transformed cells with plasmids carrying *SpGem2* and *GFP*.*SmB* that were under the control of a very strong and a medium strong *nmt1* promoter, respectively. The growth defects of cells overexpressing Gemin2 is not complemented by an augmentation in the levels of SmB in either a wild-type or a *tdSmn* genetic background (**Fig 8B**). When the same experiment was repeated using *SpSmn* instead of *SpGem2*, wild-type cells displayed a slight decline in growth whereas, as expected, *tdSmn* cells overexpressing both *GFP*.*SmB* and *SpSmn* faired better due to the increased levels of wild-type SMN. Notably, in wild-type (and *tdSmn*, data not shown) cells in which Gemin2 is upregulated, we observed a cytoplasmic accumulation of SmB in contrast to controls in which SmB was, as expected [52], predominantly nuclear (**Fig 8C**). Cells also displayed an elongated cell phenotype indicating a block in the progression through interphase of the cell cycle or cytokinesis [53]. These findings suggest that high levels of Gemin2 are toxic to cell viability through the retention of Sm proteins in the cytoplasm, a phenotype that likely represents a block in snRNP assembly.

## Discussion

In the present study, taking advantage of a higher requirement for SMN and Gemins in *Drosophila* muscle, we have delineated key Gemin3 genetic interactions within this compartment of the motor unit. Furthermore, we uncover that in *Drosophila*, increased levels of Gemin2 have a negative impact on motor behaviour and viability. Toxicity is conserved in *S*. *pombe* in which we find that retention of Sm proteins in the cytoplasm is a contributing factor.

### Delineation of key Gemin3 interactions *in vivo*

A consensus interaction map of the human SMN-Gemins complex was recently drafted by Otter and colleagues [54] based on biochemical assays. In this regard, the SMN-Gemin2-Gemin8 troika forms the complex’s backbone and recruits the remaining members in blocks. Hence, Gemin2 and SMN pull in Gemin5 and Gemin3, respectively. Gemin8 associates with Gemin4 and Gemin7, with the latter enrolling both Gemin6 and Unrip. Other significant interactions include Gemin2-Gemin7 and Gemin3-Gemin4. Although it has long been known that alterations in SMN levels have a reverberating effect on Gemin levels [38, 39, 55–58], attempts at probing for genetic interactions between SMN-Gemins complex members were unsuccessful, except for the Gemin2-SMN interaction. In this regard, mice with half the gene copy number of both *Smn* and *Gemin2* have an enhanced motor neurodegenerative phenotype that correlates with disturbed snRNP assembly [56].

In this report, we make use of a low-expressing *Gem3* ^*∆N*^ variant (*Gem3* ^*BART*^) that on its own is phenotypically benign but we find that its presence predisposes the muscle to imbalances in the levels of SMN-Gemins complex members (**Table 1**). It is thought that Gem3^∆N^, which in essence is a catalytically inactive helicase protein, antagonises the endogenous Gemin3 wild-type protein. In a model we recently proposed, high concentrations of Gem3^∆N^ compete for the partners of Gemin3 to form inactive complexes [37]. The *Gem3* ^*BART*^ hypomorph most likely creates a situation in which the level of Gemin3 interruption does not exceed a threshold beyond which motor defects become apparent. This allowed us to simultaneously manipulate the levels of query proteins to test whether the threshold is exceeded and in so doing, through synergistic epistasis, we identified a genetic interaction between Gemin3 and SMN, Gemin2 or Gemin5. It is noteworthy that synthetic lethality was at times the endpoint, a result that depended on the severity of the allele or transgene combined with the *Gem3* ^*BART*^ mutant (**Table 1**). We find it reasonable to propose that SMN, Gemin2 and Gemin5 proteins most probably constitute the core Gemin3 genetic network in *Drosophila*. Future work aimed at confirming the reported genetic interactions within the nervous system as well as the identification of additional genetic interactions is warranted.

**Table 1:** Summary of the phenotypic effects resulting from all genetic manipulations.

Genetic Interaction	Genotype: *Mef2-GAL4>*	Adult Viable	Motor Defects
***Gemin3* x *Gemin5***	*Gem3* ^*BART*^	Yes	No
	*Gem3* ^*BART*^ *X2*	Yes	Yes
	*Gem5* ^*FL*^	Yes	No
	*Gem3* ^*BART*^ *+ Gem5* ^*FL*^	Yes	Yes
	*Gem5* ^*∆N*^	Yes	No
	*Gem3* ^*BART*^ *+ Gem5* ^*∆N*^	Yes	Yes
	*Gem5-IR* ^*nan+sac*^	Yes	No
	*Gem3* ^*BART*^ *+ Gem5-IR* ^*nan+sac*^	No	N/A
	*Df(2R)exu1*	Yes	No
	*Gem3* ^*BART*^ *+ Df(2R)exu1*	Yes	Yes
***Gemin3* x *SMN***	*Gem3* ^*BART*^ *+ Smn* ^*X7*^	Yes	No
	*GFP-Smn* ^*FL*^	Yes	No
	*Gem3* ^*BART*^ *+ GFP-Smn* ^*FL*^	No	N/A
	*GFP-Smn* ^*∆6*^	Yes	No
	*Gem3* ^*BART*^ *+ GFP-Smn* ^*∆6*^	Yes	Yes
	*Flag-Smn* ^*FL*^	Yes	No
	*Gem3* ^*BART*^ *+ Flag-Smn* ^*FL*^	Semi	Yes
	*Smn-IR* ^*FL26B*^	Yes	No
	*Gem3* ^*BART*^ *+ Smn-IR* ^*FL26B*^	No	N/A
	*Smn-IR* ^*N4*^	Yes	No
	*Gem3* ^*BART*^ *+ Smn-IR* ^*N4*^	No	N/A
	*Smn-IR* ^*C24*^	Yes	No
	*Gem3* ^*BART*^ *+ Smn-IR* ^*C24*^	Yes	Yes
***Gemin3* x *Gemin2***	*Gem3* ^*BART*^ *+ Df(3L)ED4782*	Yes	No
	*Gem2* ^*FL*^	Yes	Yes
	*Gem3* ^*BART*^ *+ Gem2* ^*FL*^	No	N/A
	*Gem2* ^*∆C*^	Yes	No
	*Gem3* ^*BART*^ *+ Gem2* ^*∆C*^	Yes	Yes
	*Gem2* ^*∆N*^	Yes	No
	*Gem3* ^*BART*^ *+ Gem2* ^*∆N*^	No	N/A
	*Gem2-IR* ^*gau*^	Yes	No
	*Gem3* ^*BART*^ *+ Gem2-IR* ^*gau*^	Semi	Yes
	*Gem2-IR* ^*gau*^ *X2*	Yes	No
	*Gem3* ^*BART*^ *+ Gem2-IR* ^*gau*^ *X2*	No	N/A

### Consequences of disruptions in normal SMN-Gemins complex stoichiometry

Complete deletion of any component of the SMN-Gemins complex is incompatible with life whereas a perturbation that is restricted to the motor unit has a negative influence on motor function (reviewed in [59]). There is a plethora of evidence that links loss-of-function to defects in snRNP biogenesis with downstream consequences on splicing [29, 31–34, 60, 61]. The repercussions of a gain-of-function were unknown. In this study, we report that the upregulation of Gemin2 in a wild-type background is by itself deleterious in two model organisms whereas upregulation of SMN or Gemin5 leads to phenotypic consequences only in flies expressing the *Gem3* ^*BART*^ hypomorph (**Table 1**). In view of these findings, it is tempting to speculate that the SMN-Gemins complex is susceptible to stoichiometric changes, which means that an imbalance between members has repercussions on its substrates. Our results are in agreement with studies that highlight the interdependence of component levels within the SMN-Gemins complex. In this regard, protein levels of all Gemins except Gemin5 were found reduced in cells with low amounts of SMN, including those derived from SMA patients [38, 39, 55–58]. It is hypothesised that a disruption in the SMN-Gemins complex leads to a decrease in the protein stability of its components.

**Fig 9:**
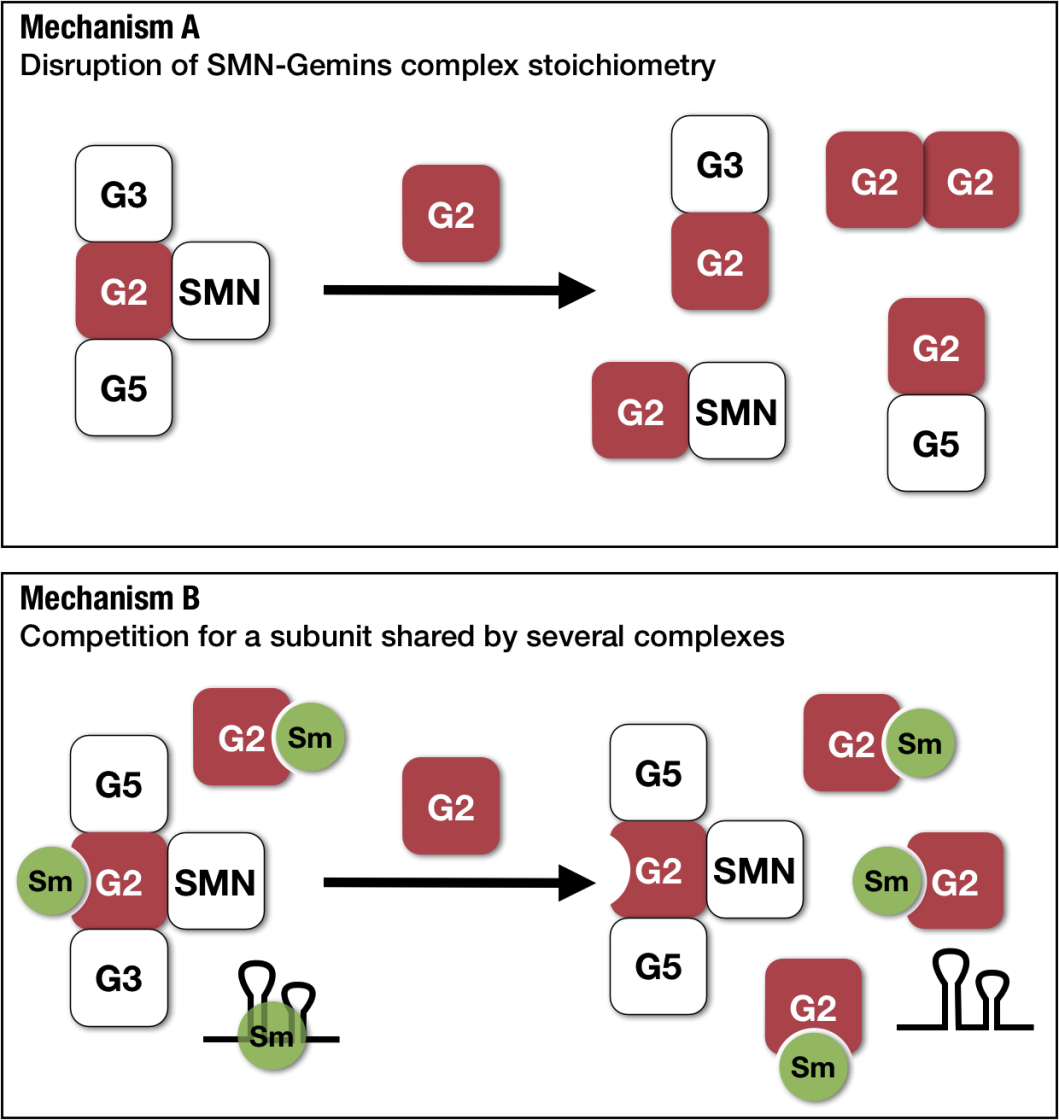
Possible mechanisms responsible for the Gemin2 overexpression phenotypes. *Mechanism A*: Gemin2 makes multiple contacts within the multi-protein SMN-Gemins complex so that its overexpression can destabilise the intact complex, thereby resulting in the formation of non-functional sub-complexes. *Mechanism B*: Sm proteins are shared subunits of snRNPs and SMN-Gemins complexes. Overexpression of Gemin2 competes for limiting amounts of Sm proteins, hence reducing their presence in other complexes. Both mechanisms predict a dysfunction in Sm core assembly on snRNAs. Abbreviations: G2, Gemin2; G3, Gemin3; and, G5, Gemin5.

Gemin2’s key role in snRNP assembly was only revealed recently through structural and biochemical studies. To this end, Gemin2 is thought to serve as the arm of the SMN-Gemins complex that captures select Sm proteins and holds them in an ordered form prior to their coupling with snRNAs. Importantly, as part of its job it prevents their assembly on unintended RNAs until the joining of an snRNA [10, 11]. Interestingly, similar to SMN and Gemin8, Gemin2 is capable of self-association, a likely requirement for its role in stabilising the SMN-Gemins complex [62]. In this context, two hypotheses can explain the toxicity associated with excess Gemin2. Since Gemin2 binds to itself and makes multiple contacts within the SMN-Gemins complex, a surplus of Gemin2 can result in partial complexes, thereby reducing the amount of the intact functional SMN-Gemins complex (**Fig 9**). Alternatively, an overabundance of Gemin2 could hijack Sm proteins, consequently reducing their capture by bona-fide SMN-Gemins complexes (**Fig 9**). Both models predict reduced cytoplasmic coupling of Sm proteins with snRNAs to form snRNPs that following assembly are normally imported in the nucleus where they function. Lending support to this prediction, we report a surplus of Sm proteins within the cytoplasm of *S*. *pombe* overexpressing Gemin2, a phenotype that is reminiscent of that reported for the dominant-negative mutant SMNΔN27 [63], and most likely indicates a cytoplasmic block in the snRNP assembly pathway. Future studies confirming that snRNP biogenesis is disrupted as well as those that distinguish between the two proposed mechanisms for a Gemin2 gain-of-function, including attempts at increasing the levels of other SMN-Gemins complex members simultaneously with Gemin2 to overturn the imbalance, are warranted.

The identification of key Gemin3 genetic interactions bodes well for future studies aimed at uncovering novel interactions. In addition to gaining insights on the function of Gemin3, and by inference, the SMN-Gemins complex, such studies might provide much needed targets for SMA therapeutic development.

## Supporting Information

S1 Fig Coupled with Gem3^BART^, reduced levels of Gemin2 in muscle lead to motor and viability defects. Knockdown of Gemin2 in muscle through the expression of either one (*Mef2-GAL4>Gem2-IR* ^*gau*^) or two (*Mef2-GAL4>Gem2-IR* ^*gau*^ *X2*) RNAi transgenes has no negative impact on both adult viability and flight ability. However, in combination with *Gem3* ^*BART*^, depending on the severity of knockdown, flies are either lethal (*Mef2-GAL4>Gem3* ^*BART*^ *+ Gem2-IR* ^*gau*^ *X2*) or semi-viable (*Mef2-GAL4>Gem3* ^*BART*^ *+ Gem2-IR* ^*gau*^). In case of the latter genotype, escapers are mostly non-fliers. Statistical significance was determined for differences between the *Mef2-GAL4>Gem3* ^*BART*^ *+ Gem2-IR* ^*gau*^ genotype, and the control *Mef2-GAL4>Gem2-IR* ^*gau*^ genotype using the unpaired t-test (****p<0.0001). Data presented are the mean ± S.E.M. of at least 4 independent experiments, and n ≥ 60 per genotype.
(TIFF)
