# Correction: Genetic Interactions between the Members of the SMN-Gemins Complex in *Drosophila*

**DOI:** 10.1371/journal.pone.0139649

**Published:** 2015-09-25

**Authors:** The PLOS ONE staff

There are multiple errors in Table 1. There is no colour separation between the 'Gemin3 x Gemin5' and 'Gemin3 x SMN' sections. The delta symbols are missing in column 2. The publisher apologizes for the error. Please see the corrected Table 1 here:

**Table 1:** Summary of the phenotypic effects resulting from all genetic manipulations.

Genetic Interaction	Genotype: *Mef2-GAL4>*	Adult Viable	Motor Defects
***Gemin3* x *Gemin5***	*Gem3* ^*BART*^	Yes	No
	*Gem3* ^*BART*^ *X2*	Yes	Yes
	*Gem5* ^*FL*^	Yes	No
	*Gem3* ^*BART*^ *+ Gem5* ^*FL*^	Yes	Yes
	*Gem5* ^*∆N*^	Yes	No
	*Gem3* ^*BART*^ *+ Gem5* ^*∆N*^	Yes	Yes
	*Gem5-IR* ^*nan+sac*^	Yes	No
	*Gem3* ^*BART*^ *+ Gem5-IR* ^*nan+sac*^	No	N/A
	*Df(2R)exu1*	Yes	No
	*Gem3* ^*BART*^ *+ Df(2R)exu1*	Yes	Yes
***Gemin3* x *SMN***	*Gem3* ^*BART*^ *+ Smn* ^*X7*^	Yes	No
	*GFP-Smn* ^*FL*^	Yes	No
	*Gem3* ^*BART*^ *+ GFP-Smn* ^*FL*^	No	N/A
	*GFP-Smn* ^*∆6*^	Yes	No
	*Gem3* ^*BART*^ *+ GFP-Smn* ^*∆6*^	Yes	Yes
	*Flag-Smn* ^*FL*^	Yes	No
	*Gem3* ^*BART*^ *+ Flag-Smn* ^*FL*^	Semi	Yes
	*Smn-IR* ^*FL26B*^	Yes	No
	*Gem3* ^*BART*^ *+ Smn-IR* ^*FL26B*^	No	N/A
	*Smn-IR* ^*N4*^	Yes	No
	*Gem3* ^*BART*^ *+ Smn-IR* ^*N4*^	No	N/A
	*Smn-IR* ^*C24*^	Yes	No
	*Gem3* ^*BART*^ *+ Smn-IR* ^*C24*^	Yes	Yes
***Gemin3* x *Gemin2***	*Gem3* ^*BART*^ *+ Df(3L)ED4782*	Yes	No
	*Gem2* ^*FL*^	Yes	Yes
	*Gem3* ^*BART*^ *+ Gem2* ^*FL*^	No	N/A
	*Gem2* ^*∆C*^	Yes	No
	*Gem3* ^*BART*^ *+ Gem2* ^*∆C*^	Yes	Yes
	*Gem2* ^*∆N*^	Yes	No
	*Gem3* ^*BART*^ *+ Gem2* ^*∆N*^	No	N/A
	*Gem2-IR* ^*gau*^	Yes	No
	*Gem3* ^*BART*^ *+ Gem2-IR* ^*gau*^	Semi	Yes
	*Gem2-IR* ^*gau*^ *X2*	Yes	No
	*Gem3* ^*BART*^ *+ Gem2-IR* ^*gau*^ *X2*	No	N/A
