# Supplementary figures and images for: Genetic Interactions between the Members of the SMN-Gemins Complex in Drosophila
Source: PLoS One. 2015 Jun 22;10(6):e0130974. doi: 10.1371/journal.pone.0130974 (PMC4476591; doi:10.1371/journal.pone.0130974)

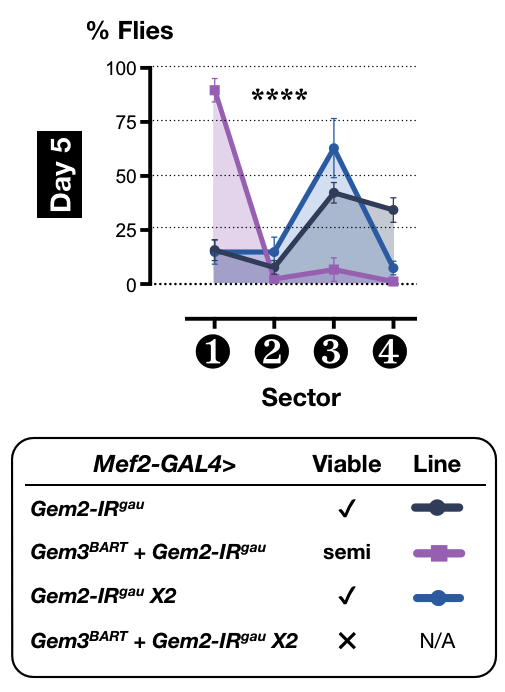

Supplement: S1 Fig — Knockdown of Gemin2 in muscle through the expression of either one (Mef2-GAL4>Gem2-IR gau) or two (Mef2-GAL4>Gem2-IR gau X2) RNAi transgenes has no negative impact on both adult viability and flight ability. However, in combination with Gem3 BART, depending on the severity of knockdown, flies are either lethal (Mef2-GAL4>Gem3 BART + Gem2-IR gau X2) or semi-viable (Mef2-GAL4>Gem3 BART + Gem2-IR gau). In case of the latter genotype, escapers are mostly non-fliers. Statistical significance was determined for differences between the Mef2-GAL4>Gem3 BART + Gem2-IR gau genotype, and the control Mef2-GAL4>Gem2-IR gau genotype using the unpaired t-test (****p<0.0001). Data presented are the mean ± S.E.M. of at least 4 independent experiments, and n ≥ 60 per genotype. (TIFF) [file pone.0130974.s001.tiff]
